# Prevalence of Cervical Cancer and Clinical Management of Women Screened positive using visual inspection with acetic acid and Cervicography in selected public sector health facilities of Manicaland and Midlands provinces of Zimbabwe, 2021

**DOI:** 10.1371/journal.pone.0294115

**Published:** 2023-11-29

**Authors:** Emmanuel Tachiwenyika, Munyaradzi Dhodho, Auxilia Muchedzi, Tafadzwa P. Sibanda, Chiedza Mupanguri, Solomon Mukungunugwa, Mutsa Mhangara, Ngonidzashe Ganje, Talent Tapera, Tendai Samushonga, Morgen Muzondo, Sithabiso Dube, Taurayi Tafuma, Byrone Chingombe, Admire Maravanyika, Tichaona Nyamundaya

**Affiliations:** 1 Technical Department, Zimbabwe Health Interventions, Harare, Zimbabwe; 2 AIDS and TB Programs, Ministry of Health and Child Care, Harare, Zimbabwe; 3 United States Agency for International Development, Harare, Zimbabwe; Newlands Clinic, ZIMBABWE

## Abstract

**Background:**

Zimbabwe has high cervical cancer (CC) burden of 19% and mortality rate of 64%. Zimbabwe uses Visual Inspection with Acetic Acid and Cervicography (VIAC) for CC screening. Manicaland and Midlands provinces recorded low VIAC positivity of 3% (target 5–25%) and treatment coverage of 78% (target = 90%) between October 2020 and September 2021.

**Objectives:**

We explored VIAC positivity rate and clinical management of clients screening positive in Manicaland and Midlands provinces.

**Methods:**

We conducted a retrospective cross-sectional study using routine VIAC and CC management data for period October 2020 to September 2021. Two samples were used, 1) a sample drawn from 48,000 women VIAC screened to measure positivity rate, and 2) a sample of 1,763 VIAC positive women to assess clinical management. Kobo-based tool was used to abstract data from facility registers, and data were analyzed using STATA 15.

**Results:**

We analyzed data for 2,454 out of 48,000 women screened through VIAC. About 82% (2,007/2,454) were HIV positive, median ages were 40 and 38 years for HIV positives and negatives respectively. Most (64% and 77%) of HIV positive and negative clients respectively were married. VIAC positivity was 5.9% and 3.4% among HIV positive and negative women screened for the first time, and 3.2% and 5.6% for repeat visits respectively. Overall, 89.1% (1,571/1,763) of VIAC positive women received treatment. Most (41%) of those treated received thermocoagulation. Overall, 43.1% of clients received treatment on VIAC day, and 77.4% within 30 days. Six-month post-treatment coverage was 3.8%.

**Conclusion:**

VIAC positivity among HIV positive women screening for the first time was 5.9%, within the expected 5–25%. Treatment coverage was high, and turnaround time from diagnosis to treatment met national standards. Post-treatment coverage was suboptimal. We recommend continued implementation of quality improvement initiatives, capacity building of clinicians, and optimization of post-treatment review of clients.

## Introduction

Cervical cancer is the fourth most common cancer among women globally, with an estimated 604, 000 new cases and 342, 000 deaths in 2020. About 90% of the new cases and deaths worldwide in 2020 occurred in low- and middle-income countries (LMIC) [1]. About 80% of cancer-related deaths in LMICs were recorded within the Sub-Saharan African region [2,3]. Women living with HIV are 6 times more likely to develop cervical cancer compared to women without HIV, and an estimated 5% of all cervical cancer cases are attributable to HIV [4]. Moreover, in all world regions, the contribution of HIV to cervical cancer falls disproportionately on younger women.

In high-income countries, programs are in place which enable girls to be vaccinated against Human Papilloma Virus (HPV), and women to get screened for cervical cancer regularly and treated adequately [5]. Screening allows pre-cancerous lesions to be identified at stages when they can easily be treated. In low-and middle-income countries, there is limited access to preventative measures and cervical cancer is often not identified until it has further advanced, and symptoms developed [6]. In addition, access to treatment of cancerous lesions e.g., cancer surgery, radiotherapy, and chemotherapy may be limited, resulting in a higher rate of death from cervical cancer in these countries [7,8].

More than 95% of cervical cancer is caused by human papillomavirus (HPV), commonly detected in cervical tumor specimens. Whilst it takes 15 to 20 years for cervical cancer to develop in women with normal immune systems, progression is faster (5 to 10 years) in women with weakened immune systems, such as those with untreated HIV infection [9]. Clinical trials and post-marketing surveillance have shown that HPV vaccines are safe and effective in preventing infections with HPV, high grade precancerous lesions and invasive cancer [10].

Cervical cancer is the most common cancer in women of all races and ages in Zimbabwe, with a burden of 19% [11]. An estimated 2,270 women are diagnosed with cervical cancer annually and the country has a high mortality rate of 64% [12]. The high HIV prevalence in Zimbabwe fuels the cervical cancer burden.

Cervical cancer screening programs have been implemented in high HIV prevalence settings using the Visual Inspection with Acetic Acid and Cervicography (VIAC) method. Zimbabwe adopted VIAC as a method of screening for cervical cancer in 2013 and VIAC remains the preferred screening method within the public sector. VIAC is an effective way to prevent cervical cancer in women aged 30–50 years, and it involves examining the cervix for changes that might lead to cervical cancer. If these changes are detected early, the cells can be eliminated before they become cancerous. The cells are usually removed using cryotherapy, thermocoagulation or Loop Electrosurgical Excision Procedure (LEEP). VIAC examinations can also reveal possible advanced cervical cancer and certain noncancerous conditions such as chronic cervicitis, pelvic inflammatory disease, and cervical polyps. VIAC and treatment using cryotherapy, thermocoagulation or LEEP has been well-studied and is endorsed by the World Health Organization (WHO). WHO recommends using this “screen-and-treat” approach as an effective, low-cost way to reduce the incidence of cervical cancer in low and medium-income countries.

The Zimbabwe Ministry of Health and Child Care (MOHCC) has an established cervical cancer prevention program that offers VIAC screening and treatment at several of the nation’s health facilities. Screening is offered at static and outreach sites, and treatment is primarily offered at referral facilities or through outreach by district health teams.

The Zimbabwe Health Interventions (ZHI) supports the MOHCC in the provision of cervical cancer screening and treatment in Manicaland and Midlands provinces of Zimbabwe as part of its HIV care and treatment program. Routine program data showed a low cervical cancer screening positivity rate of about 3% from October 2020 to September 2021. Similarly, treatment coverage for women screening VIAC positive was suboptimal, with an overall coverage of 78% during the same period. There was paucity of data to explain the low cervical cancer screening positivity rates and treatment coverages for those screening positive. We conducted this study to determine proportion of women screened VIAC positive by type of visit and HIV status, describe characteristics of women screening VIAC positive by HIV status, determine proportion of VIAC positive women who received treatment by HIV status, measure turnaround time from screening VIAC positive to receiving treatment, and determine outcomes after 6 months of follow-up for women screening VIAC positive.

## Materials and methods

### Design and participants

This retrospective, analytical cross-sectional study was conducted using routinely collected data for women screened and treated for cervical cancer. Study sites consisted of all static and outreach cervical cancer screening and treatment health facilities of Manicaland and Midlands provinces of Zimbabwe. Participants comprised of HIV positive and negative women screened for cervical cancer using VIAC between October 1, 2020, and September 30, 2021. Although the national cervical cancer screening program targets population sub-groups at high risk of developing cervical cancer such as HIV positive women, screening and treatment for cervical cancer is also provided to HIV negative clients. Study results were therefore stratified, to the extent possible, by HIV status of clients. The study was conducted between 1 January and 31 March 2022.

### Sampling methodology

A representative sample of women screening VIAC negative and positive were included in the “prevalence” sample using the following formula:

$$n^{\prime }=\frac{NZ^{2}P(1-P)}{d^{2}(N-1)+Z^{2}P(1-P)}$$

Where n = sample size, N = finite population size (48,000), Z = statistic for confidence level (1.96), P = proportion (0.19), d = precision (0.05)

Minimum sample size was 236; however, data for 2,454 women were collected to increase power. Probability proportional to size was used to determine sample sizes for facilities. Systematic sampling was used to select clients from the facility sampling frames. The cervical cancer clinical cascade sample consisted of 1,763 (out of 2,011) women who screened VIAC positive.

### Data collection tools and processes

Quantitative, secondary data were abstracted from health facility VIAC and cervical cancer management registers using a Kobo-based data abstraction tool. Variables abstracted included age, date client was screened, marital status, contraceptive method which clients used, type of clinic visit, client’s HIV status, client’s antiretroviral therapy (ART) status if HIV positive, VIAC results, histology results, type of treatment received, post-treatment review dates and treatment outcome. No personal identification data e.g., client names, physical address etc. were collected.

### Statistical analysis

Data was collected using Kobo, exported to MS Excel, and analyzed using STATA 15 (StataCorp. 2017. *Stata Statistical Software*: *Release 15*. College Station, TX: StataCorp LLC.) generating measures of central tendency and proportions.

### Ethics statement

This was an observational study which used secondary, de-identified data captured in health facility registers during routine clinical service provision to adult women. Study was covered by the Medical Research Council of Zimbabwe (MRCZ) approved non-research determination protocol (MRCZ/E/159) that provides for analysis, use, and dissemination of routinely collected program data; provision of consent was therefore waived.

### Patient and public involvement statement

This study utilized secondary cervical cancer screening and treatment data captured in health facility registers during routine health service provision. Data were abstracted directly from the registers and analysed; there was no direct contact with study participants and the public. Clients whose data were included in this study received routine health services across public sector health facilities and were not involved in the design and recruitment process as the study utilized secondary data. Findings from this study will be disseminated to the Zimbabwe Ministry of Health and Child Care (MOHCC) national, provincial and district staff, who will in-turn cascade dissemination to local and community structures including the clients.

## Results

### Demographic characteristics of women screened for VIAC

Data for 2,454 women who were randomly sampled from the 48,000 women screened for cervical cancer between 1 October 2020 and 30 September 2021 were analyzed. Table 1 summarizes demographic characteristics of participants.

**Table 1:** Demographic characteristics of participants.

Variable	HIV Positive (N = 2,007)	HIV negative (N = 445)	Unknow HIV status (n = 2)
Age Median (IQR)	40(IQR 34–46)	38(IQR 28–46)	
**Marital Status** Divorced Married Separated Single Widowed	50(2%) 1,279(64%) 90(4%) 245(12%) 343(17%)	5(1%) 344(77%) 9(2%) 51(11%) 36(8%)	1 1

Eighty-two percent (2,007/2,454) were HIV positive with a median age of 40 years (IQR 34–46); 445 women were HIV negative with a median age of 38 years (IQR 28–46). Most of the HIV positive (64%) and HIV negative (77%) women were married.

### VIAC screening by visit type

About 75,1% (1,507/2,007) and 82.4% (371/445) of HIV positive and negative women were screening for the first time respectively (Table 2). Overall VIAC positivity rate was 5.4% and 3.4% among HIV positive and negative women respectively. VIAC positivity rate was 5.9% and 2.7% among HIV positive and negative women screening for the first time respectively, and 3.2% and 5.6% among women repeat screened.

**Table 2:** Characteristics of women VIAC screened in selected facilities of Manicaland and Midlands provinces by visit type and HIV status, October 2020 to September 2021.

Variable	HIV Positive (N = 2007)	HIV negative (N = 445)	Unknow status (n = 2)
Type of Visit New Post treatment Referral Repeat	1507(75.1%) 23(1.1%) 4(0.2%) 473(23.6%)	371(82.4%) 3(0.7%) 0 71(16%)	1 1
VIAC results-All Positive Negative Suspicious	110(5,4%) 1888(94.1%) 9(0.5%)	15(3.4%) 426(95.7%) 4(0.9%)	2
VIAC Results-New Visit Positive Negative Suspicious	(n = 1507) 89(5.9%) 1413(93.8%) 4(0.3%)	(n = 371) 10(2.7%) 359(96.8%) 2(0.5%)	
VIAC Results-Post Treatment visit Positive Negative Suspicious	(n = 23) 3(13%) 20(86.9%) 0	(n = 3) 1 2 0	
VIAC Results-Referral visit Positive Negative Suspicious	(n = 4) 3 1 0		
VIAC Results-Repeat visits Positive Negative Suspicious	(n = 473) 15(3.2%) 454(95.1%) 4(0.9%)	(n = 71) 4(5.6%) 58(91.5%) 2(2.8%)	

### Characteristics of women screening VIAC positive

A total of 1,763 (out of 2,011) women who screened VIAC positive in the period under review were included in the cervical cancer clinical cascade. About 89% (1,584/1,763) were HIV positive, and median ages were 38 and 34 years for the HIV positive and negative respectively (Table 3). The median age at first sexual encounter was 19 and 18 years for HIV positive and negative women respectively. The most common family planning method used was the pill, with 39% and 49% of HIV positive and negative women using it respectively.

**Table 3:** Characteristics of women screening VIAC Positive in selected facilities of Manicaland and Midlands provinces, October 2020 to September 2021.

Variable	HIV positive (N = 1,584)	HIV negative (N = 179)
Age [Median (IQR)]	38(IQR 31–43)	34(IQR 27–41)
Age at first sex [Median (IQR)]	19(IQR 18–20)	18(IQR 17–20)
Marital Status Divorced Married Separated Single Widowed	58(3.7%) 951(60%) 109(6.9%) 240(15.1%) 226(14.2%)	5(2.8%) 144(80.5%) 8(4.5%) 18(10.1%) 4(2.2%)
Parity [Median (IQR)]	2(2–4)	2(1–4)
Commonly Used Family Planning method Depo-Provera Implant None Pill Other	238 (15%) 134(8%) 480(30.3%) 620(39.1%) 112(7%)	29(16.2%) 18(10.1%) 34(18.9%) 88(49.1%) 10(5.5%)

Of the VIAC positive women included in the analysis, 80% of HIV positives and 86% of HIV negatives were identified during initial screening. About 16.5% (262/1,584) of HIV positive and 11.7% (21/179) of HIV negative VIAC positive clients were identified during repeat visits. About 2.4% (39/1,584) and 1.7% (3/179) of HIV positive and negative VIAC positive clients were identified during the 6-month post-treatment visit; the remainder (0.8% and 0.6% of HIV positive and negative VIAC positive clients respectively) were referrals from other facilities. Most of the VIAC positive women were using the pill for family planning (39% for HIV positive and 49% for HIV negative); 30.3% and 18.9% of HIV positive and negative women respectively were not on any family planning method.

### Treatment of VIAC positive clients

Overall, 89.1% (1,571/1,763) VIAC positive clients received treatment. Out of the 1,571 who were treated, most (41%) received Thermocoagulation followed by LEEP (37.9%) and Cryotherapy (8.5%). The remainder received Hysterectomy (0.2%), knife cone biopsy (0.2%) and other treatment (1.3%) (Table 4).

**Table 4:** Uptake of treatment among women who screened VIAC positive by treatment type.

Variable	HIV positive (N = 1,584)	HIV negative (N = 179)
Thermocoagulation	652 (41.2%)	70 (39.1%)
LEEP	628 (39.7%)	41 (22.9%)
Cryotherapy	113 (7.1%)	37 (20.7%)
Hysterectomy	3 (0.2%)	1 (0.6%)
Knife Cone Biopsy	1 (0.1%)	2 (1.1%)
Other	20 (1.2%)	3 (1.7%)
Not Treated	167 (10.6%)	25 (14%)

About 10.6% and 14% of HIV positive and negative women respectively did not get treatment.

### Turnaround time from screening VIAC positive to treatment

Overall, 43.1% of clients received treatment on the same day of screening VIAC positive and 77.4% within 30 days (Table 5).

**Table 5:** Turnaround time from screening VIAC positive to treatment by modality.

Treatment Modality	Treated on the same day of screening VIAC positive (N = 678)	Treated within 30 days of screening VIAC positive (N = 1,216)
Thermocoagulation	541 (79.8%)	652 (53.6%)
LEEP	42 (6.2%)	419 (34.5%)
Cryotherapy	91 (13.4%)	139 (11.4%)
Hysterectomy	2 (0.3%)	2 (0.2%)
Knife Cone Biopsy	0	1 (0.1%)
Other	2 (0.3%)	3 (0.2%)
Overall	43.1% (678/1,571)	77.4% (1,216/1,571)

About 79.8% of clients who received treatment on the day of screening VIAC positive and 53.6% of those who received treatment within 30 days of screening positive had Thermocoagulation. Only 6.2% of VIAC positive women who received treatment on the day of diagnosis had LEEP.

### Post treatment coverage of VIAC positive clients

The six-month post-treatment coverage for eligible women who screened VIAC positive was 3.8% (31/815).

## Discussion

Overall, VIAC positivity rate was higher among HIV positive (5.9%) than HIV negative (3.4%) women (p<0.05), reflecting the higher risk of cervical cancer among immunocompromised individuals. Our study provides evidence that VIAC positivity among HIV positive women screened for the first time in Manicaland and Midlands provinces of Zimbabwe (5.9%) was within the expected range of between 5 to 25% among this population subgroup [13]. VIAC positivity rate was however lower among HIV positive women who were re-screened (3.2%). These findings suggest that the low positivity (3%) recorded from routine HIV care and treatment data was a result of not disaggregating data by visit type; including women who were re-screened in the analysis therefore lowered the positivity rate.

Results from this study were dissimilar to those reported in a study conducted by Fallala et al at one facility in Bulawayo, Zimbabwe which recorded overall VIAC positivity of 10.8% [14]. The Bulawayo study was conducted in one specialist referral health facility using data for the period 2010 to 2012. It is possible that those who were screened would have been referred for cervical cancer related conditions hence the high positivity rate. Additionally, this assessment was conducted before VIAC was adopted as a national cervical cancer screening method in 2013; screening coverage was still low, and most women were being screened for the first time, hence the relatively high positivity rate. Results from our analysis were similar to findings from a study by Gabaza et al conducted in Harare, Zimbabwe which recorded a VIAC positivity of 6.5% [15]. This study used data for the period 2012 to 2016 which coincided with national roll out of VIAC in Zimbabwe. Our results were also similar to findings from a Mozambique study by Moon T et al which recorded VIAC positivity of 8% [16]; a 5.1% VIAC positivity rate was recorded in a Malawi study by Msyamboza et al [17].

Treatment coverage for VIAC positive women was high (89.6%), marginally falling short of the WHO-treatment target of 90% as set out in the global strategy to accelerate elimination of cervical cancer [18]. This treatment coverage was higher than the 76% recorded in Zambia between 2010 and 2019 [19] and 43% recorded in Malawi between 2011 and 2015 [17]. The high treatment coverage recorded in our study can be attributed to program innovations including targeted outreach to hard-to-reach areas by district health teams. LEEP treatment camps organized by district health teams ensured treatment for eligible clients closer to their areas of residence. Additionally, bus-fares were provided for needy clients requiring treatment, and where feasible clients were transported using project vehicles. The 10.4% of VIAC positive clients who did not receive treatment can be attributed to delays in clients accessing specialist services including LEEP which could only be offered by trained medical doctors. Some LEEP eligible clients identified at primary level facilities had to wait for an outreach LEEP camp by district medical team or needed to travel to district and provincial hospitals where the service was available. Results from this analysis showed that only 6.4% of LEEP eligible women received treatment on the same day of screening VIAC positive.

Turnaround time (TAT) from screening VIAC positive to treatment was relatively short, with 43% and 45% of HIV positive and negative clients respectively receiving treatment on the day of screening. A majority i.e., 77% and 83% of HIV positive and negative women respectively received treatment within 30 days of screening VIAC positive. These turnaround times align with the Zimbabwe national cervical cancer treatment standards which recommend treatment for precancerous lesions within 30 days of screening [20]. The TAT from screening VIAC positive to treatment with thermocoagulation and cryotherapy was the shortest, with a median of 0 days, demonstrating fidelity in the implementation of the "screen-and-treat" approach. Thermocoagulation and cryotherapy contributed over 90% (79.8% and 13.7% respectively) of VIAC positive women treated on the day of screening. For clients who received LEEP, the median TAT was 20 days; this relatively longer TAT was largely a result of resource and logistical challenges in getting clients to treatment centers or hosting LEEP camps.

The 6-month post treatment follow up for VIAC positive women was suboptimal at 3.8%. This can partly be a result of suboptimal documentation of this visit in the current facility-held registers due to poor understanding of documentation requirements. Additionally, health care workers might not have been emphasizing the need for clients to return for this important clinical review.

### Limitations

Our findings are subject to some limitations. First, study participants may not be representative of all women in Zimbabwe given the study was limited to 2 out of 10 provinces. Second, the study utilized secondary data captured in health facility paper registers. There was a risk that some entries would not be complete either because of poor documentation in registers or poor data capture during abstraction. We mitigated these limitations by using a digital, Kobo-toolbox-based data abstraction tool with validation rules. This enabled data cleaning during collection and improved completeness of abstracted data. All entries with incomplete variables were cross-checked with health facility staff and where this was not possible and missing variables were substantial, these entries were not abstracted. Despite these potential limitations, our study findings represent VIAC positivity rates, treatment coverage and turnaround time from screening VIAC positive to treatment among HIV positive and negative women accessing cervical cancer screening and treatment services in public sector health facilities in Zimbabwe.

## Conclusion

The VIAC positivity rate among HIV positive women screening for the first time in Midlands and Manicaland provinces was 5.9%, in line with the expected 5–25% for this population group. Treatment coverage for VIAC positive clients was high, and turnaround time from diagnosis to treatment met the national standards. The 6-month post-treatment review of VIAC positive clients was suboptimal. Healthcare workers (HCW) providing cervical cancer management services should optimize post-treatment review of VIAC positive clients through physical and virtual tracking and tracing, targeted outreach to hard-to-reach areas and provision of bus-fare for needy clients. HCW should harmonize cervical cancer screening and review visits for HIV positive women with medicine pick-up or viral load testing to optimize service uptake. Additionally, deliberate efforts should be made in identification of HIV positive women due for post-treatment review during routine HIV review visits. Implementation of continuous quality improvement projects across all health facilities should be optimized to identify root causes for cervical cancer screening and treatment gaps and implementation of innovations to address them. The monitoring and evaluation team within Ministry of Health and Child Care should prioritize revision of the VIAC register to enable generation of unique client identification number that facilitates tracking of clients coming for repeat visits; the register should also have data elements to capture client outcomes such as lost to follow-up, referrals, and treatment completion.

## Supporting information

S1 Data (XLSX)

S2 Data (XLSX)

S1 File (PDF)
