# Supplementary material for: Prevalence of Cervical Cancer and Clinical Management of Women Screened positive using visual inspection with acetic acid and Cervicography in selected public sector health facilities of Manicaland and Midlands provinces of Zimbabwe, 2021
Source: PLoS One. 2023 Nov 29;18(11):e0294115. doi: 10.1371/journal.pone.0294115 (PMC10686478; doi:10.1371/journal.pone.0294115)
Supplement: S1 File — (PDF) [file pone.0294115.s003.pdf]

Telephone: 791792/791193
Telefax: (263) - 4 - 790715
E-mail: mrcz@mrcz.org.zw
Website: http://www.mrcz.org.zw

Medical Research Council of Zimbabwe
Josiah Tongogara / Mazoe Street
P. O. Box CY 573
Causeway
Harare

CONTINUING APPROVAL

REF: MRCZ/E/159 **05 April 2022**

Tichaona Nyamundaya
Zimbabwe Health Interventions
65 Whitwell Road, Borrowdale West
Harare

RE: Zimbabwe HIV Care and Treatment (ZHCT) Project Non-Research Determination of Its Routine Project Data Documentation and Dissemination

Thank you for the application for review of Research Activity that you submitted to the Medical Research Council of Zimbabwe (MRCZ). Please be advised that the Medical Research Council of Zimbabwe has **reviewed**and **approved**your application to continue conducting the above titled study.

This approval is based on the review and approval of the following documents that were submitted to MRCZ for review:-

a) Completed MRCZ Form 102
b) Progress report

- **APPROVAL NUMBER** **: MRCZ/E/159**

This number should be used on all correspondence, consent forms and documents as appropriate.

- **TYPE OF MEETING** **: EXPEDITED**
- **EFFECTIVE APPROVAL DATE** **:10 February, 2022**
- **EXPIRATION DATE** **:09 February, 2023**

After this date, this project may only continue upon renewal. For purposes of renewal, a progress report on a standard form obtainable from the MRCZ Offices should be submitted three months before the expiration date for continuing review.

- **SERIOUS ADVERSE EVENT REPORTING**: All serious problems having to do with subject safety must be reported to the Institutional Ethical Review Committee (IERC) as well as the MRCZ within 3 working days using standard forms obtainable from the MRCZ Offices or website.
- **MODIFICATIONS**: Prior MRCZ and IERC approval using standard forms obtainable from the MRCZ Offices is required before implementing any changes in the Protocol (including changes in the consent documents).
- **TERMINATION OF STUDY**: On termination of a study, a report has to be submitted to the MRCZ using standard forms obtainable from the MRCZ Offices or website.
- **QUESTIONS**: Please contact the MRCZ on Telephone No. (04) 791792, 791193 or by e-mail on mrcz@mrcz.org.zw

Other

- Please be reminded to send in copies of your research results for our records as well as for Health Research Database.
- You're also encouraged to submit electronic copies of your publications in peer-reviewed journals that may emanate from this study.
- The study can be conducted with verbal consent.

Yours Faithfully

………………………………………………………

MRCZ SECRETARIAT
FOR CHAIRPERSON
MEDICAL RESEARCH COUNCIL OF ZIMBABWE

MEDICAL RESEARCH COUNCIL OF ZIMBABWE
05 APR 2022
APPROVED
20 CAMBRIDGE ROAD, AVONDALE, HARARE
